# The Impact of an Evidence-Informed Spinal Cord Injury Activities of Daily Living Education Manual (SADL-eM): Protocol for a Randomized Controlled Trial

**DOI:** 10.2196/30611

**Published:** 2022-07-22

**Authors:** Moussa Abu Mostafa, Nicola Ann Plastow, Maggi Savin-Baden, Birhanu Ayele

**Affiliations:** 1 Division of Occupational Therapy Faculty of Medicine and Health Sciences Stellenbosch University Cape Town South Africa; 2 Occupational Therapy Department Hamad Rehabilitation Hospital Khanyouniss Occupied Palestinian Territory; 3 School of Education University of Worcester London United Kingdom; 4 Division of Epidemiology and Biostatistics Faculty of Medicine and Health Sciences Stellenbosch University Cape Town South Africa

**Keywords:** occupational therapy, educational intervention, activities of daily living, spinal cord injury, clinical trials

## Abstract

**Background:**

Spinal cord injury (SCI) is a catastrophic injury associated with functional loss and life-threatening complications. Many people with SCI in the Gaza Strip of Palestine are discharged from inpatient rehabilitation to the community while still lacking many daily life skills. This randomized controlled trial (RCT) seeks to test the impact of the Spinal Cord Injury Activities of Daily Living Education Manual (SADL-eM)—an evidence-based occupational therapy patient educational intervention—on rehabilitation outcomes.

**Objective:**

The proposed trial aims to evaluate the SADL-eM intervention compared with standard treatment among people with SCI.

**Methods:**

This is a parallel RCT with two study arms: intervention and control. A total of 90 patients treated in inpatient rehabilitation settings will be randomly allocated to two study groups. Both groups will receive standard care. The intervention group will also use the SADL-eM with their treating occupational therapist during rehabilitation. The SADL-eM is a comprehensive activities of daily living (ADL) educational tool that was codeveloped with people with SCI and stakeholders across Gaza. The self-report version of the Spinal Cord Independence Measure will be used on admission (ie, baseline measure) and after 6 weeks as the primary outcome measure. Secondary outcomes include the third version of the Spinal Cord Independence Measure, the Private Religiousness Practices Scale, the Organizational Religiousness Short-Form, additional ADL domains covered by the education manual, and adherence to the intervention. The effect of the intervention will be determined using repeated-measures analysis of variance.

**Results:**

This study will be conducted from April 2021 through December 2022, with results expected to be available in January 2023.

**Conclusions:**

If the SADL-eM is demonstrated as clinically effective, this will have significant implications for occupational therapy interventions in low- and middle-income countries.

**Trial Registration:**

ClinicalTrials.gov NCT04735887; https://clinicaltrials.gov/ct2/show/NCT04735887

## Introduction

### Background

Spinal cord injury (SCI) describes the damage to the spinal cord resulting from trauma (eg, stab wound), disease (eg, transverse myelitis), or degeneration (eg, due to a tumor or infection, such as tuberculosis). An SCI can result in paralysis of the extremities and trunk below the level of lesion that determines residual motor and sensory dysfunction [1]. All people with SCI have the right to participate in activities and occupations that are meaningful and purposeful, and that enable them to reach their desired potentials. However, many people with SCI in low- and middle-income countries (LMIC) are discharged from inpatient rehabilitation to the community while still dependent during many activities of daily living (ADLs). These ADLs include basic self-care activities, such as bathing, dressing, toileting, eating, and mobility [2]. Limited access to community-based rehabilitation services means that inpatient rehabilitation services need to be as effective as possible before discharge. In addition, patients need access to relevant information when they return home and continue their recovery journey. This paper describes a randomized controlled trial (RCT) study protocol that seeks to test the impact of the Spinal Cord Injury Activities of Daily Living Education Manual (SADL-eM), a contextually relevant education tool for people with SCI living in the Gaza Strip of Palestine.

### Patient Education Following Spinal Cord Injury

People with SCI have many information needs after injury. Patient education is one affordable and accessible strategy that may enhance the effectiveness of rehabilitation for people with SCI in LMIC. Health professionals, such as occupational therapists, are a preferred source of information for health education seekers about SCI [3,4]. Education for patients with SCI aims to develop knowledge, skills, attitudes, and behaviors to maintain health and well-being, prevent secondary complications, and enhance active life participation after SCI [5]. Caregivers of people with SCI reported that health education is associated with less burden of disability. The most common topics covered include general health-related issues, home adaptation, assistive devices, and financial aspects [4]. Letts and colleagues [6] suggested that body anatomy and functioning topics should be presented early in the admission phase, while other topics like community integration can be presented at later phases of rehabilitation, so that people with SCI become progressively more independent.

Effective SCI education should be accessible and motivating in terms of information and how it is presented [3]. Educational interventions should provide support, for example, access to online support groups, consultations with rehabilitation professionals, and opportunities to ask questions. SCI educational interventions should also be cost-effective and up to date, and should meet the needs of the diversity of people accessing rehabilitation services [6]. It is also important that SCI education for adults promote autonomy and is guided by adult learning theory to facilitate the active roles of people with SCI [7]. Continuing postdischarge care and linking to community resources and the health care system are critical components of SCI education. Staff availability and patient readiness are also two important concerns for effective patient education. Time allocated for patient education can be inadequate due to patient treatment workload and staffing shortages. Decreased motivation and interest on the part of the patient during rehabilitation are other barriers to patient education [8].

Despite widespread guidance on education for patients with SCI, little research has been conducted on the effectiveness of education focused on ADLs for people with SCI [3,9]. A recent systematic review of clinical trials of ADL educational interventions identified only three interventions in four publications [10]. None of these significantly improved participation, although a meta-analysis showed that two of the interventions had a positive effect on the performance of ADLs, mobility (ie, wheelchair ambulation and transfers), and prevention of secondary complications, and resulted in a decrease in doctors’ visits. In addition, all three interventions were delivered in high-income countries.

The first intervention included in that systematic review was the clinical practice guidelines for Preservation of Upper Limb Function Following Spinal Cord Injury, an ADL educational program focused on wheelchair ambulation and transfers. It involved two separate and specific forms: one for the therapist and one for the patient. The patient form was designed to be accessible, appropriate for novice adults without a previous medical background, and organized into modules. The therapist form was more complex regarding the level of information and was suitable for health professionals with a medical educational background [11]. To meet different learners’ styles and preferences, different educational formats were used, including interactive discussions, printed handouts, pictures, and videos illustrating wheelchair propulsion and transfer skills to take home after discharge [11].

In the second intervention, the Peer Mentoring Programme, people with SCI acted as mentors and educators to people with newly acquired injuries. The peer mentors saw their mentees in person or communicated with them by phone during daily life scenarios within the first week of admission to active inpatient rehabilitation. The mentors then monitored the health status of their mentees using the Medical Complications Tracking Form; provided education relevant to ADL, prevention of complications, and incontinence issues; and initiated referrals to health care professionals if needed [12].

The third intervention, the Needs Assessment and Goal Planning Programme (NAGPP), is a comprehensive rehabilitation tool for everyday clinical use that was used as an ADL educational program. The Needs Assessment Checklist (NAC) is a part of the NAGPP and is used to evaluate and compare rehabilitation needs and outcomes. Therefore, the NAC reflects the patient’s perception of his or her needs, choices, and priorities. The NAC was administered twice by a key worker who had the responsibility of coordinating the Rehabilitation and Goal Planning Programme: the first after beginning the active rehabilitation program and the second on admission to the predischarge rehabilitation ward. The key worker had the responsibility of coordinating the goal planning system with other members of the multidisciplinary team, and the patient was responsible for establishing and identifying needs, clarifying goals, and specifying targets [13].

More recently, Ziba and colleagues [14] evaluated the effect of a self-care educational program on the quality of life for people with SCI. Their study demonstrated that self-care program training was effective in improving the quality of life of people with SCI. Furthermore, Zarei and colleagues [15] investigated the positive effect of the education of patients with SCI and its contribution to satisfaction with marital sex life in men with SCI. These two clinical trials demonstrated the association between self-care education and satisfaction gained by improved levels of functionality and performance of ADLs. In their scoping review of health education by peers for people with SCI, Chaffey and Bigby [16] addressed another aspect of quality of life and satisfaction in their finding that people with SCI perceived their participation in peer education programs as supportive. In addition, education that includes peers helps people with SCI explore their potential, promotes self-confidence, and improves health outcomes.

### Context of This Study

Palestine is characterized as one of the LMIC [17]. In 2016, Palestine registered 504 new cases of SCI, while the total cases with SCI in Palestine were 21,989. The incidence of SCI in Palestine was 90 cases per million people, while the prevalence was 6130 cases per million people; these data are relevant to Palestine. People with SCI in the Gaza Strip are predominantly young men, Muslim, living in family homes, and unemployed [18]. People with SCI requiring inpatient rehabilitation in the Gaza Strip complete their medical management in a secondary health care facility. Once medically stable, they are referred and admitted to an inpatient rehabilitation setting. Inpatient medical rehabilitation is provided in three clinical settings, which provide a short stay of 6 to 8 weeks. Rehabilitation focuses on preventing secondary complications and optimizing function. SCI rehabilitation aims to promote the participation of people with SCI in meaningful occupations of choice [19].

Similar to people with SCI in other LMIC, people with SCI in Gaza are frequently discharged prematurely from inpatient rehabilitation to the community, while lacking essential self-care skills, such as toileting and safe transfers [1]. This places them at higher risk of life-threatening complications. Community-based rehabilitation is not widely available. Assistive technology is often required by people with SCI to facilitate occupational participation. However, only 5% to 15% of people with SCI in LMIC have access to assistive technology [17].

Barriers to effective rehabilitation for people with SCI in Gaza include timely admission to a suitable rehabilitation setting, inadequate stays in inpatient rehabilitation, insufficient learning of daily life skills during inpatient rehabilitation, and lack of access to proper assistive technology. Moreover, many patients are added to the waiting list before admission. These barriers drive the need to develop evidence-based interventions during inpatient care that will improve rehabilitation outcomes, such as performance of ADLs [10].

There is little information about preferred educational formats on the part of people with SCI due to limited research targeting this topic. Some authors regard the internet as the best option, due to low cost and wide reach [20]. This is not always the case. Most people with SCI in LMIC are unemployed young men who have financial limitations and, therefore, cannot afford to pay for the internet and advanced technology, such as cell phones and iPads. We believe that a low-cost, paper-based manual format will enable improved access to information during inpatient rehabilitation, as well as when patients return to their community.

In 2018 we began a project to develop and evaluate the effectiveness of patient education for rehabilitation services in Gaza. We used participatory action research methods to develop an ADL education manual—the SADL-eM—that could promote occupational therapy education for people with SCI in the Gaza Strip, Palestine [21]. The aim of this study is to evaluate the impact of using the SADL-eM as an information tool during occupational therapy with people with SCI who are undergoing inpatient rehabilitation for an SCI in the Gaza Strip, Palestine. By improving patient education, this study may improve rehabilitation outcomes and patients’ involvement in their treatment during their short stay in one of three inpatient rehabilitation settings available in the Gaza Strip. An effective educational intervention may also enhance the quality of care and reduce the cost of health care services in the long term.

### Research Question

The research question for this study is as follows: Does the implementation of the SADL-eM delivered to patients with SCI in an inpatient rehabilitation setting in the Gaza Strip improve independence in ADL and participation over 6 weeks?

### Primary Objectives

The primary objectives for this study are as follows:

1. To compare the effect of the education manual, relative to usual care, on self-reported changes in ADL from admission to 6 weeks postadmission, using the self-report version of the Spinal Cord Independence Measure (SCIM-SR) patient-rated measure.
2. To compare the effect of the education manual, relative to usual care, on observed changes in ADL from admission to 6 weeks postadmission, using the third version of the Spinal Cord Independence Measure (SCIM-III) clinician-rated measure.
3. To compare the effect of the education manual, relative to usual care, on religious practice, using the Private Religiousness Practices Scale and Organizational Religiousness Short-Form.
4. To compare the effect of the education manual, relative to usual care, on the domains of ADL covered by the education manual, including driving and community mobility, personal device care, health management and maintenance, ablution, and Islamic prayer, using questions developed for this research.

### Secondary Objectives

The secondary objectives for this study are as follows:

1. To determine the significance of biographical variables (ie, address zone, age, gender, highest level of education, marital status, employment status, monthly income, level of SCI, and type of SCI: complete or incomplete), the American Spinal Injury Association Impairment Scale score, and dysfunction on changes in ADL performance.
2. To measure adherence to the intervention using the therapist-report questionnaire regarding SADL-eM adherence, which will be developed for this purpose.

### Research Hypotheses

The null hypothesis for this study is as follows: there is no ADL outcome difference between the SADL-eM and the standard care delivered to inpatients with SCI in a rehabilitation setting.

The alternative hypothesis for this study is as follows: the implementation of the SADL-eM delivered to inpatients with SCI in a rehabilitation setting has a significant impact on their ADL.

## Methods

### Study Design

**Figure 1:**
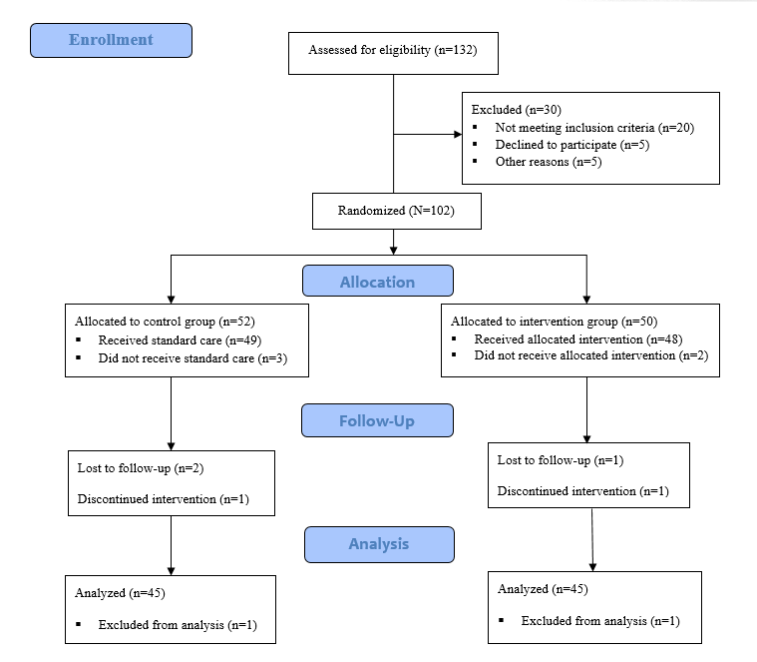
Study flow diagram according to the CONSORT (Consolidated Standards of Reporting Trials) statement.

Figure 1 shows the study flow diagram. This is a parallel RCT with a pretest and a posttest to evaluate a hypothesis of a cause-and-effect relationship. This study uses the CONSORT (Consolidated Standards of Reporting Trials) statement as the proposed standard for the reporting of parallel-group RCTs [22].

### Participants and Recruitment

Participants in the study will be inpatients with SCI from the Gaza Strip, of both genders, with any cause or type of SCI, and aged 18 to 65 years. They will be recruited from the group of admitted inpatients in one of the three rehabilitation settings in the Gaza Strip, Palestine: Hamad Rehabilitation Hospital, El-Wafa Rehabilitation Hospital, and Al-Amal Rehabilitation Hospital. Readmitted patients will be included in the study if they are eligible. Over the first 3 days of admission of each person with SCI in any of the three rehabilitation facilities, a clinical research supervisor will recruit study participants based on the study eligibility criteria. Inclusion and exclusion criteria are listed in Textbox 1.

**Inclusion and exclusion criteria.**

**Inclusion criteria**

- Confirmed spinal cord injury (SCI) diagnosis by computed tomography or magnetic resonance imaging report
- The American Spinal Injury Association Impairment Scale (ASIA): categories A, B, and C
- Aged between 18 and 65 years old
- Stable medical condition
- Time elapsed after SCI is not more than 6 months
- Minimum time of stay in inpatient rehabilitation unit is 6 weeks
- Active involvement in rehabilitation program
- Sufficient comprehension (ie, reading and writing) of the Arabic language

**Exclusion criteria**

- Unconfirmed diagnosis
- Patients who have communication or cognitive disorders, such as global aphasia and memory deficit
- Patients with a disturbed level of awareness, such as being in a coma or experiencing lethargy
- Time elapsed since SCI is more than 6 months
- ASIA: categories D and E
- Unstable medical condition
- Patients who have other causes of disability in addition to SCI, such as stroke or amputation
- Aged less than 18 years old or more than 65 years old
- Time of stay in inpatient rehabilitation unit is less than 6 weeks
- Inactive involvement in rehabilitation program
- Patients with a progressive disease or a psychiatric condition that would interfere with active participation in the rehabilitation program
- Patients with cardiovascular contraindications
- Persons who become ambulatory during the inpatient period
- Persons with complete tetraplegia caused by an SCI at the C4 level or above
- Persons on mechanical ventilation

### Informed Consent

Each eligible participant who agrees to participate in the study will sign a written consent form before being included any of the study procedures or interventions. This consent form will be kept in a locked filing cabinet that will only be accessible by the research team.

### Sample Size Calculation

We aim to recruit 90 participants to demonstrate a small effect caused by the intervention, with a postintervention mean difference of 15% between the intervention and control groups. The mean scores of the SCIM-III and the SCIM-SR at enrollment are expected to be 30% for both groups; at postintervention, these are expected to be 65% for the intervention group and 50% for the control group. A sample size of 90 participants, with 45 in each group, is sufficient for 80% power, a 5% margin of error, a CI of 95%, and an SD of 20%.

### Blinding

This study uses an assessor-blinded data collection method. The study process conceals the intervention group identity from outcome assessors after participants’ treatment assignment through randomization to minimize possible bias on the part of the outcome assessors, which could influence the reliability of study results. Moreover, therapists involved in the study and participants in the intervention group will be informed to refrain from sharing any part of the manual or included information with other patients or staff.

### Randomization

The study sample of 90 SCI individuals will be randomized into two groups using a simple randomization approach, where an allocation list will be generated using the Random Number Generator online program. The two groups are as follows: conventional therapy group (n=45) and intervention (ie, SADL-eM) plus conventional therapy group (n=45). Eligible participants will be assigned to one study group according to the generated list.

### Study Setting

Hamad Rehabilitation Hospital, El-Wafa Rehabilitation Hospital, and Al-Amal Rehabilitation Hospital are three inpatient rehabilitation settings that are part of the private health sector in the Gaza Strip of Palestine and provide comprehensive rehabilitation services. Their rehabilitation teams include physical rehabilitation doctors, rehabilitation nurses, physiotherapists, occupational therapists, speech therapists, clinical dietitians, psychologists, social workers, orthoptists, and community-based rehabilitation workers.

### Feasibility Study

Feasibility studies are preliminary studies with small samples that investigate whether the main study can be conducted under the given conditions. RCTs usually use the feasibility study design to improve the trial’s feasibility and avoid crucial barriers [23]. A total of 15 participants with SCI of any cause and type, aged 18 to 65 years old, and with a previous inpatient rehabilitation experience will participate in the feasibility study. They will be selected from the 2019-2020 inpatient list from the Hamad Rehabilitation Hospital. They will complete the SCIM-SR. A well-trained research assistant who is an occupational therapist will administer the biographical data collection tool, the SCIM-III, the Private Religiousness Practices Scale, the Organizational Religiousness Short-Form, and data collection regarding the domains of ADL covered by the education manual, including driving and community mobility, personal device care, health management and maintenance, ablution, and Islamic prayer. The patients and the research assistant will comment on the tools employed and point out any problems with the tests’ instructions, instances where items are not clear, and formatting and other typographical errors and issues. Each question that measures performance of and participation in ADL will be analyzed using Cronbach α to test and improve reliability.

### Activities of Daily Living Education Manual for People With SCI: The SADL-eM

Both study groups will receive usual care. In addition, each participant in the intervention group will receive a copy of the SADL-eM. The SADL-eM was developed by the authors following a systematic review and user-led development project. A team of 54 subject matter experts collaborated in a participatory action research project to develop the manual. To our knowledge, the SADL-eM is the first comprehensive SCI ADL educational tool available in Arabic [21].

The SADL-eM includes three elements essential to the intervention: knowledge, skills, and advice. The manual is made up of six detailed sections, including an introduction and the following five chapters: (1) Rehabilitation Team, (2) ADL, (3) Assistive Devices, (4) Home Environment Adaptation, and (5) Knowledge Guide. The SADL-eM uses text and illustrative pictures that were carefully selected for contextual relevance. The manual is simple and easy to use, and is suitable for people with a nonmedical background. However, good Arabic-language comprehension and the ability to read and write is mandatory for users. The purpose of the SADL-eM is to serve as a treatment tool during inpatient rehabilitation [21].

A clinical research supervisor will provide each participant in the intervention group with a hard copy of the SADL-eM and will explain the purpose of the manual and how to use it. The manual will be reviewed by the treating occupational therapist who received training on the use of the SADL-eM (ie, a 6-hour training on the minimum standards of the use of the SADL-eM for SCI education) and by the person with SCI during their treatment sessions. During occupational therapy sessions, the therapist will refer to the relevant chapter of the manual and answer the participant’s queries regarding the content of the manual. The therapist will also indicate to the participants which parts of the manual are not relevant to them, based on their gender, driving status, or level of SCI.

According to the minimum standards of SADL-eM use for SCI education, the therapist will include the manual in as many SADL-eM educational sessions as required, but not less than three 15-minute sessions per week. The minimum level of clinical experience required of each treating occupational therapist will be 2 years. Good Arabic-language comprehension and the ability to read and write is mandatory for both therapists and patients. Commitment and adherence to the SADL-eM minimum standards are prerequisites and will be assured during the consenting of participants.

The participants will keep the manual to review after sessions and after discharge from the inpatient rehabilitation setting. The research assistant who performs data collection, the clinical research supervisor, and the occupational therapy team will be invited for an informational session prior to data collection in each of the participating settings. The informational session will focus on the study purpose, recruitment, eligibility criteria, use of the SADL-eM, roles, bias reduction, and blinding. The use of data collection tools will take place in a separate session with the research assistant. The principal researcher will answer all questions from the research team during informational sessions and during the research study.

For the purpose of this study, the SADL-eM will be provided during face-to-face individual occupational therapy sessions without any constraints regarding the treatment area. The face-to-face delivery of the SADL-eM does not require any infrastructure or special equipment.

The number of sessions, time allocated, and frequency of sessions will be determined by each therapist and participant to ensure that the treatment is person centered, given that the minimum standards of the SADL-eM implementation are met. Determinants may include a participant’s capabilities, potential, and progress during treatment as well as the scope of the service, such as time allocated for each patient, therapist-to-patient ratio, and frequency of inpatient sessions. The therapist and the participant will continue to use the manual during treatment sessions until they decide there is no need for further use of the manual or the participant is discharged. The treating occupational therapists will retain records of any change or modification of the SADL-eM administration. The researchers will conduct a pilot study in a similar sample of 15 people with SCI to improve the use of the intervention and data collection tools.

Nonadherence to medical treatment in clinical trials of medical efficacy may obscure the data that are collected and disrupt the findings that are obtained [24]. The steps to ensure adherence to the intervention during the study include clearly defining the intervention, measuring adherence, and then comprehensively reporting adherence to the intervention. Attendance records collected by the therapist (ie, time, duration, and intensity of treatment and elements covered by the manual) and the therapist-report questionnaire regarding SADL-eM adherence will be used to measure and report adherence to the SADL-eM intervention.

### Data Collection

Table 1 outlines the schedule of the enrollment tasks, interventions, and assessments that occurred at the study time points.

**Table 1:** The schedule of enrollment tasks, interventions, and assessments.

Study tasks				Study time point										
				Enrollment: Apr 2021		Allocation: Day 0		Postallocation						
								Day 1		Day 42 (week 6)		Day 43		Day 44
**Enrollment tasks**														
	Eligibility screen		✓^a^											
	Informed consent		✓											
Admission to study						✓								
**Interventions**														
	Intervention A^b^						✓		✓					
	Intervention B^c^						✓		✓					
**Assessments**														
	**Baseline variables**													
		Patient education			✓									
		ADL^d^			✓									
	**Outcome variables**													
		SCIM-SR^e^			✓						✓			
		SCIM-III^f^			✓						✓			
	**Other data variables**													
		The Private Religiousness Practices Scale			✓						✓			
		Organizational Religiousness Short-Form			✓						✓			
		SADL-eM^g^ adherence therapist-report questionnaire											✓	

^a^A checkmark indicates that the task, intervention, or assessment was performed at the indicated time point.

^b^Inpatient rehabilitation with standard care.

^c^Health education using the SADL-eM.

^d^ADL: activities of daily living.

^e^SCIM-SR: self-report version of the Spinal Cord Independence Measure.

^f^SCIM-III: third version of the Spinal Cord Independence Measure.

^g^SADL-eM: Spinal Cord Injury Activities of Daily Living Education Manual.

Participants’ recruitment and admission to the study will continue until the desired sample size is reached. The allocation sequence will be concealed from patients, therapists, and assessors. The data regarding exposure to the educational intervention will be collected by a separate research assistant (ie, the research coordinator) by phone and will be concealed from the assessors. Each occupational therapy department will assign four therapists to follow up the trial cases: two for the intervention group and two for the control group. All the staff in the occupational therapy department, the participants, and the assessors will be asked to refrain from discussing the trial and intervention material between them. The importance of concealing any information until the end of the study will be explained. Study participants will not be allowed to switch between occupational therapists or study arms. They will be asked not to participate in other educational interventions, peer or external, related to their rehabilitation or seek treatment outside their trial. The head of the occupational therapy department within each rehabilitation setting will supervise participants and the assigned occupational therapists in both study arms to reduce contamination bias, such as sharing intervention content with the control group, sharing similar time and space of occupational therapy sessions, and exchange of therapists.

Contamination bias due to already-existing background or receipt of additional educational interventions will be measured within each arm of the study using the chi-square test to compare the progress means. However, no case will be eliminated from statistical analysis due to contamination bias.

The study will adopt approaches to prevent the loss of participants to follow-up by making questionnaires as easy to complete as possible, providing incentives in the form of a free copy of the education manual for the control group after completing the study, and explaining the importance of the study [25].

The researchers will use the same outcome measures throughout the study and within both study arms. Biographical data will be collected by a research assistant once on admission of the participant and after they have provided consent to participate. ADL data collection from each participant in the study will take place in a face-to-face interview at two time points: once on admission before being provided with the education manual and again 6 weeks later. This data will be collected using the following: (1) the patient-administered SCIM-SR and (2) the research assistant–administered SCIM-III, Private Religiousness Practices Scale, Organizational Religiousness Short-Form, and domains of ADL covered by the education manual, including driving and community mobility, personal device care, health management and maintenance, ablution, and Islamic prayer. The data will be used to answer the research question and test the study hypothesis.

The validity of the experimental study can be threatened by history, selection, and maturation [26]. The validity of this study’s findings will be enhanced by concealing the intervention group using a blinded assessor throughout the study. Sampling and contamination bias will be minimized through randomization and assuring the freedom of participation and anonymity of participants.

Objective data collected in clinical settings using valid and reliable tools are essential for evaluating the impact of an intervention [27]. The validity and reliability of the data collected and the results of the study will be improved using standardized measurement tools, such as the SCIM-SR and the SCIM-III (Table 2 [27-31]).

**Table 2:** Summary of data collection tools.

Data collection tool	Items, n	Score range; interpretation	Scale	Language version	Administration	Psychometric properties
Biographical data collection tool	24	N/A^a^	Nominal, ordinal, and interval	English	Assessor reported	Developed by the researchers to be used in this study
SCIM-SR^b^ [27]	17	0-100; a higher score indicates better function	Ordinal	English; will be translated into Arabic	Patient reported	Good reliability: Pearson correlation coefficients and ICCs^c^ of the total and subscale scores are above 0.7
SCIM-III^d^ [27,28]	17	0-100; a higher score indicates better function	Ordinal	English	Assessor reported	Good reliability: Cronbach α=.70-.78; κ coefficient=0.64-0.84; Pearson correlation coefficients and ICCs of the total and subscale scores are 0.84-0.94
The Private Religiousness Practices Scale [29]	4	4-27; a lower score is better	Ordinal	English	Assessor or patient reported	Acceptable reliability: Cronbach α=.70
The Organizational Religiousness Short-Form [29,30]	2	2-18; a higher score is better	Ordinal	English	Assessor or patient reported	Acceptable reliability: Cronbach α=.70-.76
The SADL-eM^e^ adherence therapist-report questionnaire [31]	8	N/A	Nominal and interval	English	Occupational therapist reported	Developed by the researchers to be used in this study

^a^N/A: not applicable; this measure does not give scores as outcomes.

^b^SCIM-SR: self-report version of the Spinal Cord Independence Measure.

^c^ICC: intraclass correlation coefficient.

^d^SCIM-III: third version of the Spinal Cord Independence Measure.

^e^SADL-eM: Spinal Cord Injury Activities of Daily Living Education Manual.

### Primary Outcome Tool

The SCIM-SR evaluates the ability of a person with SCI to perform specified activities independently, with assistance or with assistive devices, from the perspective of the people with SCI. Research supports the criterion validity of SCIM-SR [27]. The tool is not available in Arabic so the first author will translate the SCIM-SR to Arabic by forward-backward translation and test the usability of the Arabic version in the feasibility study.

### Secondary Outcome Tools

The biographical data collection tool captures demographic and socioeconomic data, including age, gender, marital status, level of education, employment, monthly income, accommodation, diagnosis, and cause and type of SCI.

The SCIM-III evaluates the ability of a person with SCI to perform specified activities independently, with assistance or with assistive devices, from the perspective of the health care provider. It requires 30 to 45 minutes to complete the assessment by observation and about 20 minutes by interview [27]. The SCIM-III has been evaluated in multiple countries, including in the Middle East, and it appears to be resistant to cross-cultural differences [32]. The tool is not available in Arabic so the English version will be used [28].

The Private Religiousness Practices Scale measures the frequency of an individual’s involvement in religious behaviors: prayer, religious attendance (ie, attending a church or mosque), reading the Bible or Quran, and watching religious programs on television [29]. The tool is not available in Arabic so the English version will be used [30].

The Organizational Religiousness Short-Form measures the involvement of the individual with a formal public religious institution, such as a church or mosque [29]. The tool is not available in Arabic so the English version will be used [30].

The SADL-eM adherence therapist-report questionnaire is based on the Morisky Medication Adherence Scale. This tool measures any change to the supplied SADL-eM in terms of the content of the intervention, topics, described techniques, mode, format, and degree of individualization of the intervention [31]. The tool keeps a record of educational sessions by day, duration, intensity, and elements covered. This English version of this tool will be used.

### Ethical Considerations

Ethical approval was obtained from the Stellenbosch University Human Research Ethics Committee (HREC project ID: 1635) and the Helsinki Committee for Ethical Approval (PHRC/HC/689/20). This trial was registered at ClinicalTrials.gov (NCT04735887). At recruitment, participants will be provided with comprehensive information to explain the trial and their participation. Patients’ questions will be answered by the researcher honestly and as fully as possible. Participants’ consent to be included in the study will be recorded in writing. All the data and the signed consent forms will be securely stored during and after study completion in a locked cabinet on hospital premises, up to 5 years after publication. Participants will be free to withdraw at any time. Participant anonymity and confidentiality of information collected for the research will be assured in the consent form and sustained throughout the study. The study is unlikely to result in any harm, risk, or discomfort to participants. The participants in the control group will receive a copy of the SADL-eM at the end of the study.

### COVID-19 Considerations

The treating occupational therapists and research assistant will follow the required COVID-19 precautions published by the World Health Organization and applied by each facility [33]. This includes the use of medical disposable gowns, disposable latex gloves, face shields, surgical masks, and hand hygiene using 70% alcohol or alcohol gel. These supplies will be provided by each facility as requirements of usual treatment.

### Data Analysis Plan

Captured data will be coded and reviewed by the authors for clarity and completion. A spreadsheet will be prepared by and then loaded into SPSS software (version 21; IBM Corp) for analysis. To ensure study reliability, the first author will perform data entry, the research assistant will check the data entry using the original forms, and the clinical research supervisor will check the data for missing data and entry errors. During analysis, missing data will be replaced by the means, for continuous or discrete variables, or modes, for ordinal or nominal variables.

To describe participants’ characteristics (eg, age, gender, level of injury, and completeness of injury), descriptive statistics, such as frequency, percentage, and mean and SD, will be used for normally distributed data. Median and IQR will be used for continuous variables that are not normally distributed. The Shapiro-Wilk test will be used to test data distribution normality [34].

We will then use the repeated-measures ANOVA and linear mixed models to compare the mean scores of the primary outcome tool (ie, SCIM-SR) and the secondary outcome tools (ie, the SCIM-III, the Private Religiousness Practices Scale, and the Organizational Religiousness Short-Form) to test the research hypothesis. Linear mixed models have an advantage in dealing with missing values and provide fixed and random effects [25]. Repeated-measures ANOVA assumptions will be tested using the Mauchly test of sphericity [34]. If this test is nonsignificant (*P*>.05), we will assume that the data meet the assumption of sphericity, similar to the homogeneity of variance for between-group ANOVA. Violation of the sphericity assumption will require correction using either the Greenhouse-Geiger correction (ε<.75) or the Huynh-Feldt correction (ε>.75). The effect size will be calculated using Cohen *d* [35]. Corallo et al [36] provided accepted benchmarks for SCIM-III outcomes for clinicians and researchers: 12% for tetraplegia and 43.3% for paraplegia. Table 3 summarizes the statistical analysis tests intended to be used in this study.

**Table 3:** Summary of statistical analysis plan.

Study objective	Analysis plan
To compare the effect of the education manual, relative to usual care, on self-reported changes in ADL^a^ from admission to 6 weeks postadmission, using the SCIM-SR^b^ patient-rated measure	Repeated-measures ANOVA and linear mixed models to assess differences between intervention and control groups using the ADL data
To compare the effect of the education manual, relative to usual care, on observed changes in ADL from admission to 6 weeks postadmission, using the SCIM-III^c^ clinician-rated measure	Repeated-measures ANOVA and linear mixed models to assess differences between intervention and control groups using the ADL data
To compare the effect of the education manual, relative to usual care, on religious practice using the Private Religiousness Practices Scale and the Organizational Religiousness Short-Form	Repeated-measures ANOVA and linear mixed models to assess differences between intervention and control groups using the ADL data
To compare the effect of the education manual, relative to usual care, on the domains of ADL covered by the education manual, including driving and community mobility, personal device care, health management and maintenance, ablution, and Islamic prayer, using questions developed for this research	Repeated-measures ANOVA and linear mixed models to assess differences between intervention and control groups using the ADL data
To determine the significance of biographical variables (ie, address zone, age, gender, highest level of education, marital status, employment status, monthly income, level of SCI^d^, and type of SCI: complete or incomplete), the ASIA^e^ score, and dysfunction on changes in ADL performance	McNemar and chi-square tests for nominal variables; *t* tests and Mann-Whitney *U* tests for interval variables
To measure the adherence to the intervention using the SADL-eM^f^ adherence therapist-report questionnaire, which will be developed for this purpose	Descriptive statistics, such as frequency and percentage

^a^ADL: activities of daily living.

^b^SCIM-SR: self-report version of the Spinal Cord Independence Measure.

^c^SCIM-III: third version of the Spinal Cord Independence Measure.

^d^SCI: spinal cord injury.

^e^ASIA: American Spinal Injury Association Impairment Scale.

^f^SADL-eM: Spinal Cord Injury Activities of Daily Living Education Manual.

This is an RCT that will be conducted in clinical settings (ie, rehabilitation hospitals) where contamination bias is evident. Therefore, we followed the intention-to-treat (ITT) principle to assess outcomes based on the treatment group and not based on the intervention. By using the ITT principle, we aim to avoid potential bias due to exclusion of ineligible cases and prevent attrition bias when evaluating the intervention [26].

### Protocol Amendments

The authors will request approval for important protocol modifications, such as changes to eligibility criteria, outcomes, and analyses, from the HREC of Stellenbosch University under the regulations of the Declaration of Helsinki. Deviations from this protocol will be reported when publishing or presenting the trial outcomes.

## Results

This is an approved RCT that was registered in 2020. Data collection for the internal pilot study started in April 2021. Between April 2021 and March 2022, 32 cases were eligible and included in the study. The majority were males (n=23, 72%), and the rest were females (n=9, 28%). Each of the intervention and control groups included 16 cases. Trial data collection is expected to continue until December 2022. Results are expected to be available in January 2023.

## Discussion

### Overview

The anticipated main finding of this study is that the use of a codeveloped, contextualized ADL education manual during inpatient occupational therapy rehabilitation will improve performance of ADL for people with SCI living in Gaza, Palestine, in comparison to usual care. The study findings will provide us with important information about the performance of ADL among people with SCI, based on widely used data collection tools (ie, the SCIM-SR and the SCIM-III), as well as participation in other important aspects of religious life (ie, the Private Religiousness Practices Scale and the Organizational Religiousness Short-Form) and community life (ie, driving and community mobility, personal device care, and health management and maintenance). The findings will also illustrate adherence on the part of people with SCI and health professionals to the educational intervention.

Providing occupational therapy patient education that meets the needs of people with SCI aims to improve their ADL performance and optimize their rehabilitation outcomes [10]. The SADL-eM is an evidence-informed codeveloped manual that supports a client-centered approach and aims to teach people with SCI how to handle their ADL after injury, during the inpatient rehabilitation phase, and when living at home. We hope that the SADL-eM will promote occupational participation, health and well-being, quality of life, and community inclusion among people with SCI in LMIC, where medical interventions are evaluated based on their outcomes and cost-efficacy [37].

Inpatient SCI education is designed to promote participation [10]. A systematic review identified three health and participation educational interventions. Although many ADL domains were not addressed in these interventions, there is evidence that SCI education that is focused on ADL optimizes rehabilitation outcomes [10]. More recent studies indicated increased demand for health education, addressed wider ADL domains, used new educational interventions, and highlighted the importance of previous interventions. People with SCI have also demonstrated their interest in topics such as community resources, home modifications, assistive devices, and driving rehabilitation [38].

A specific educational mobile app was evaluated among men with SCI and showed positive results in the marital sex life of men with SCI [39]. Mortenson et al [40] evaluated stakeholders’ perspectives on the development of a functional mobile app to facilitate self-management skills needed to prevent secondary complications following recent SCI during inpatient rehabilitation. Stakeholders focused on individualized tools, targeting goals, and increasing participation.

Self-care and peer education are basic interventions in health and participation education. A self-care educational program was found to be significant in inpatient rehabilitation and improved the quality of life of people with SCI [16]. Peer education has been used for more than two decades in people with SCI and it is gaining more interest. People with SCI perceived peer education as a means of “opening closed doors” and “going back to life.” This viewpoint was attributed to the support and experience that the peer educators provided to people with SCI [16]. Recently, Carney et al [41] initiated a large project to develop the International Spinal Cord Injury/Dysfunction Education Basic Data Set. This database is accessible by service developers and researchers, service providers, and service users.

The use of a randomized controlled study design is a strength of this study. High-quality and well-controlled clinical trials are essential to developing effective interventions for people with SCI [10,11]. An additional strength is the involvement of people with SCI and a wide range of health professionals involved in rehabilitation following SCI, from all three rehabilitation services in Gaza, in the development of the education manual. Co-construction with service users and service providers means that the voices of important stakeholders are represented. Consequently, the educational intervention is culturally relevant to people with SCI living in Gaza. In particular, the manual addresses the unique participation needs of Muslim people with SCI. Furthermore, the intervention advocates for the right to health information as well as participation in a wide range of activities and occupations that are meaningful to people with SCI in Gaza.

If the SADL-eM is demonstrated to be clinically effective, it will have significant implications for occupational therapy research and practice in LMIC, where limited resources are allocated for SCI rehabilitation, community-based rehabilitation, and assistive devices. Furthermore, people with SCI experience many forms of occupational injustice in LMIC [17]. Future studies need to address the possible long-term maintenance of improvement associated with the use of the SADL-eM, and the impact of SADL-eM use during rehabilitation on other aspects, such as quality of life.

### Limitations

We were unable to identify any study in LMIC that provides information about ADL performance among people with SCI measured by the SCIM-SR and SCIM-III. However, there are a number of potential limitations to the proposed study. This RCT employs strict inclusion criteria to improve the reliability of findings. Meanwhile, the incidence of SCI in the Gaza Strip is relatively low. Therefore, data collection is expected to continue for 12 to 14 months (April 2021-December 2022), during which different types of bias may be introduced. For example, sampling bias may occur due to a possible high rate of withdrawals and dropouts from the study. Familiarity of health professionals and people with SCI with the intervention is likely to create contamination bias. While the risk of maturity bias may occur due to age and long length of stay in the inpatient rehabilitation settings, exposure bias may occur due to different lengths of stay in the inpatient rehabilitation settings [42].

The intervention used in this RCT concurs with physical distancing that is currently required in clinical settings due to COVID-19. Considering this, it is safe and compatible with infection control guidelines [33,43].

### Conclusions

The findings of the proposed study will highlight the importance of a culturally relevant educational intervention in people with SCI in LMIC and will, therefore, pave the road for more constructive and creative solutions to the lack of resources in these countries. This will encourage the shift in rehabilitation services toward more just and inclusive approaches. Future dissemination and implementation efforts will be critical to ensure that this cost-effective and easily accessible intervention is used to promote participation. The SADL-eM will be made freely available on the Stellenbosch University website in Arabic, with a summary in English. The study results will be further disseminated through conference presentations.

## Abbreviations

ADL: activities of daily living
CONSORT: Consolidated Standards of Reporting Trials
HREC: Human Research Ethics Committee
ITT: intention-to-treat
LMIC: low- and middle-income countries
NAC: Needs Assessment Checklist
NAGPP: Needs Assessment and Goal Planning Programme
RCT: randomized controlled trial
SADL-eM: Spinal Cord Injury Activities of Daily Living Education Manual
SCI: spinal cord injury
SCIM-III: third version of the Spinal Cord Independence Measure
SCIM-SR: self-report version of the Spinal Cord Independence Measure

## Data Availability

Anonymized data will be available for peer review or quality appraisal from the corresponding author. Any other data sharing will require approval from the authors (MAM, NAP, and MSB), ethical approval from the requesting party, and approval from the HREC of Stellenbosch University.
